# Expectations, realities and challenges of good dementia care for Turkish migrants in Germany: a qualitative interview study

**DOI:** 10.1186/s12913-026-14728-3

**Published:** 2026-05-15

**Authors:** Ela Nadine Yüzgülen, Sabine Salloch, Zümrüt Alpinar-Segawa

**Affiliations:** Hannover Medical School, Institute for Ethics, History and Philosophy of Medicine, Carl- Neuberg-Str. 1, 30625 Hannover, Germany

**Keywords:** Culturally sensitive care, Dementia care, Germany, People with a Turkish migration background

## Abstract

**Background:**

As the number of people with a migration background in Germany continues to rise, it is becoming increasingly critical to provide dementia care that addresses their needs. This study investigates the specific needs and barriers faced by people with a Turkish migration background in accessing dementia-related services, as well as the challenges experienced by healthcare professionals. By directly comparing family caregivers with and without a Turkish migration background, and by including healthcare professionals with and without a migration background, this study addresses an important research gap and explores how cultural narratives shape perceptions of good dementia care.

**Methods:**

We conducted semi-structured, in-depth interviews with 11 professionals and 15 family caregivers of Turkish and German descent in Germany from November 2023 to June 2024. Data were analyzed using qualitative content analysis to identify key themes related to care experiences, service accessibility, and cultural perceptions of good dementia care.

**Results:**

Family caregivers with a Turkish migration background often had limited knowledge of dementia, experienced cultural stigma, and preferred to care for their relatives at home due to strong familial and cultural expectations as well as financial barriers. They made little use of formal support services, often due to language barriers, fear of social judgment, and lack of culturally appropriate care options. In contrast, caregivers without migration background were generally better informed, more open to external help, and more likely to access professional care services. These differences were also influenced by the educational level of the interviewees, with higher levels of education linked to better knowledge and greater use of support structures. Interviews with professionals supported these findings. The need for diagnosis tools for foreign-born people and the need for culturally sensitive care based on biographical information was emphasized.

**Conclusions:**

This study highlights the urgent need for culturally sensitive dementia care in Germany with the aim of addressing the knowledge gaps, language barriers, and ethical conflicts encountered by people with a migration background. Incorporating individuals’ cultural needs into healthcare can improve access, alleviate the burden on families, and ensure that care is addressed to their needs.

**Supplementary Information:**

The online version contains supplementary material available at 10.1186/s12913-026-14728-3.

## Background

The number of people with a migration background living in Germany is constantly increasing [1], resulting in growing diversity in the cultural, religious and linguistic terms. People of Turkish origin in Germany have a long history of immigration since the 1960s. With a population of 2.85 million, Turkey is the largest country of origin for people with a migration background. This means that 3.5% of Germany’s population has a Turkish migration background [2]. As a result of demographic changes, the need for dementia related services and culturally sensitive care among people with a Turkish migration background are becoming increasingly important in Germany [3, 4]. According to estimates, 137.300 people with dementia and with a migration background live in Germany. This is the highest number in Europe and is probably due to Germany having the largest population of people with a migration background over the age of 65 [5].

Studies suggest that older people with a migration background are more likely to experience health problems and require more care compared to those without. This is often linked to physically demanding work, psychosocial stress and barriers in accessing healthcare services [3, 6–8]. The first generation of Turkish migrants who came to Germany as guest workers experienced stressful and disadvantaged situations due to factors such as working conditions, income levels and unemployment [3, 6]. It has been argued that people with a migration background have certain care needs due to their experience of migration and cultural differences, a point that is particularly important in the case of dementia [4, 9]. Currently, there is limited data available on the care needs of people with a migration background, particularly in the context of dementia, and the available data often related to the overall German population. One reason for the lack of migration-specific data is that the target group is difficult to engage in research due to the stigmatization and tabooing of the syndrome and the community is “hard to reach for research participation due to factors such as shame about the disease and past experiences of discrimination” [3, 10, 11].

Access to care is a key prerequisite for good health. However, people with a migration background often encounter various access barriers. These include stigma, marginalization, language skills and a lack of knowledge regarding health services, support and care offers. For example, studies conducted with people with a Turkish migration background showed that they lack knowledge about dementia and its progress [3, 11, 12]. Additionally, a lack of information about access to care options, cultural norms such as responsibility of the family members for care, and the fear of stigmatization, as well as possible language barriers, restrict access to care support services. Conversely, there is often a lack of cultural sensitivity among professionals, which can have a significant impact on patients’ experiences [4, 13]. Access to culturally sensitive facilities and the integration of people with a migration background into activities in retirement homes are major structural barriers [14].

Many established services are less accessible for people with a migration background due to such migration-specific hurdles [15]. Even though there are first attempts of implementing culturally sensitive healthcare services and migration-specific projects on dementia in Germany, there is still a need for better access to healthcare services [16].

Expectations of good care are culturally shaped [4]. However, culturally sensitive services specifically for people with a migration background are currently lacking [15, 17]. The aim of culturally sensitive care is to meet the needs of people with a migration background by taking their perspectives and needs into account. Among other things, religious, cultural and linguistic aspects play a significant role here.

Legal provisions in Germany also shape the care situation and access to health services for people with a Turkish migration background. According to the General Equal Treatment Act (Allgemeines Gleichbehandlungsgesetz, AGG) (§ 1) [18], institutions and service providers (including healthcare services) are obliged to ensure equal access to diagnostics, counselling, and care for all individuals, regardless of their origin, language, or religion [19]. However, due to loopholes in the law, this is not always the case. Long-term care insurance arrangements may be very important for people with a Turkish migration background, for example, if they return to Turkey to receive cross-border care services. Under the current provisions, entitlement to the long-term care allowance is maintained only during permanent residence in the European Union (EU) or the European Economic Area (EEA) countries or Switzerland [20]. As Turkey is not one of these countries, the German-Turkish Social Security Agreement does not provide long-term care insurance coverage for individuals residing in Turkey. This means that individuals who move to Turkey are no longer entitled to German long-term care insurance. Consequently, families who wish to move their relatives with dementia to Turkey or continue their care there often lose their right to receive care assistance [20, 21].

Most elderly people with a Turkish migration background are cared for by their families, as this is considered a filial duty [3, 11, 12, 22, 23]. Additionally, there is a reluctance to use professional healthcare services and institutional facilities due to cultural and language barriers, as well as a lack of migration-sensitive care [22]. This situation can lead to significant financial difficulties and have a major impact on the choice of care for a family member, whether at home or at a care facility [7, 24]. Given these facts, long-term care insurance regulations are unfortunately not only a legal issue but also a socio-ethical challenge, as they may increase structural barriers to migrant-sensitive care arrangements [19].

With this exploratory comparative study, we aim to find out (a) the needs of people with a Turkish migration background in accessing dementia care, and (b) the challenges experienced by professionals in caring for and counseling people with a migration background in regard to dementia-related healthcare services. As we want to identify culturally determined differences in the attitudes, expectations and perceptions in managing care, we also included family caregivers of people with dementia without a migration background in our study. We were also interested in participants’ general perceptions of good dementia care. Therefore, the analysis aims to explore the influence of various cultural narratives on the understanding of good dementia care.

To the best of our knowledge, no studies have been conducted in Germany that directly compare the challenges experienced by people with a Turkish migration background with those experienced by those without a migration background with regard to dementia. Nor have any studies examined the perspectives of professionals with and without a migration background together. Therefore, this study addresses a significant research gap and contributes to the literature on migration and dementia care.

## Methods

A qualitative approach has been chosen to gain authentic insights into stakeholder perspectives on dementia care for Turkish migrants in Germany. From November 2023 to June 2024, eleven semi-structured face-to-face and telephone interviews with professionals were conducted covering a wide range of expertise in dementia, aging and care with a focus on intercultural work and counseling for seniors with and without migration background. The professionals either had a Turkish migration background (*n* = 7) or were without Turkish migration background (*n* = 4). In addition, fifteen interviews with family members of people with dementia with Turkish descent (*n* = 7) or German descent (*n* = 8) were conducted from February 2024 to June 2024. The study protocol was approved by the Ethics Committee of Hannover Medical School (No. 10976_BO_K_2023).

### Recruitment process and sample composition

We recruited professionals with experience of working for seniors in intercultural settings and specializing in ageing, dementia and dementia care, such as doctors in neurology and psychiatry (*n* = 4), psychologists (*n* = 2) nurses (*n* = 2) and social workers (*n* = 3). There were four male and seven female interview participants. The interviewed professionals were identified via internet research and snowball sampling. In all professional groups, at least one person with Turkish migration background was included. The years of experience of the professionals interviewed ranged from 10 to 35 years. Three of the interviewees did not indicate exact years of experience (see Table 1).

Family members of a person with dementia (*n* = 15) were recruited via different channels. Posters and flyers were prepared in Turkish and German and publicly disseminated at collaborating memory clinics, as well as via emails sent to self-help organizations in Lower Saxony, Germany. In order to reach more people with a Turkish descent, mediators and counsellors, who are working in special organizations with a focus on migration and interculturality, societies and help-centers for migrants were contacted. The recruitment also included self-help group meetings. During recruitment, the researchers met difficulties when contacting family caregivers with Turkish descent. People’s hesitation to participate in a study on dementia as a topic played a big role. Their hesitation was mostly due to their worry concerning sufficient anonymization, which likely led them to refrain from participating in the study.

In our sample composition, we tried to achieve a socio-demographical variety with family caregivers (see Table 2). All interviewed family members were involved (more or less) with the care of their family members with dementia. The extent of involvement in care varied, according to participants’ self-description, between extensive levels of care or rather low personal involvement. One participant was not a family member, but was very close to the family and was involved actively in taking care of the person with dementia. Two of the participants’ family members with dementia had passed away before the start of the study. The interviewed family caregivers have an age range from 22 to 81 with the mean 59,6. The recruited family members included 13 females and 2 males reflecting a gender bias in informal care.

**Table 1:** Background information on interviewed professionals (*n* = 11)

Years of experience*¹	Gender
10	Male
17	Male
27	Male
N/A*²	Female
35	Male
15	Female
N/A*²	Female
8	Female
29	Female
N/A*²	Female
12	Female

*¹ Experience in working related to ageing, dementia and intercultural work for seniors with and without migration background, care and counseling

*² No available data on years of experience

**Table 2:** Socio-demographic data of the interviewed family members (*n* = 15)

Characteristic	*n*	%
Gender		
Female	13	86.7
Male	2	13.3
Age group		
<40	1	6.2
40–49	4	25.0
50–59	4	25.0
60–69	1	6.2
≥70	5	31.2
Migration background		
Turkish migration background	7	46.7
No migration background	8	53.3
Relation to PwD		
Daughter/Son	8	53.3
Spouse	1	6.7
Other relative	5	33.3
Close acquaintance (non-family)	1	6.7
Employment status		
Employed	9	60.0
Retired / pensioned	5	33.4
Other	1	6.7
Education		
Secondary education	5	33.3
Vocational training	5	33.3
University degree	3	20.0
Other	2	13.3

### Study design

The interviews were conducted according to semi-structured interview guidelines (see Supplement 1). The guideline was developed to examine topics related to (a) experiences and knowledge about dementia and its management; (b) understanding of good dementia care; (c) assessment of dementia-related healthcare services; (d) experiences of challenging or unpleasant situations; and (e) understanding culturally sensitive care and its assessment in practice.

### Data collection and analysis

All interviewees were informed clearly about the procedure, recording, transcription, anonymization, and publications of the results. They provided written informed consent after having read a description of the study. Interviewees were free to withdraw from the study at any point in time. Family members were offered 25 Euros for their participation in the study. The interviews were conducted by Zümrüt Alpinar Segawa (ZAS) or Ela Nadine Yüzgülen (ENY) separately either in Turkish or in German. Both interviewers have a migration background in Turkey, with ZAS being the first generation to migrate to Germany and ENY being the third generation to live in Germany with a migration background. Sabine Salloch (SaS) contributed to the data analysis. She has no migration background. The interviews with the professionals lasted between 29 min and 83 min with the mean duration of 48 min. The interviews with family members lasted between 27 min and 65 min with the mean duration of 42,3 min. All interviews were audio recorded by the interviewer and transcribed verbatim by a professional external service provider (for German) and by ZAS for Turkish. 18 interviews were conducted in German, 8 interviews were conducted in Turkish. Transcriptions were fully anonymized to protect the interviewees’ anonymity. Interview transcripts were analyzed according to key principles qualitative content analysis (Kuckartz 2019; Rädiker and Kuckartz 2020). For the analysis, specific key terms and topics were categorized in relation to the research question. Based on these key terms, the anonymized interview texts were coded and afterwards reviewed and discussed by both authors until a consensus was reached. For list of codes, see Table 3. Coding and analysis were supported by MAXQDA as a software.

**Table 3:** List of Codes based on key terms

Category	Sub-categories/Coding	Description
Claim of support with dementia care	Provision of support	The code refers to statements on the use of assistance. This can be, for example, the support of the family through home care services or the requirement of a nursing home for people with dementia. It includes statements regarding actively seeking and asking advice from a doctor and approaching further medical measures.
Information & Counseling	Provision of information	The code refers to statements about receiving information. It includes statements regarding the lack of information. This could be, for example, a lack of information from the doctor or a lack of information about financial support from the health insurance company.
	Counseling	The code refers to statements about receiving counseling. It includes statements regarding the lack of receiving counseling.
Perceptions of dementia	Perceptions of dementia	The code is used for the statements referring to the differences between persons with a Turkish migration background and those without a migration background (i.e., Germans) in their perceptions of and approaches towards dementia.
Provision of good dementia care	Definition of good dementia care	The code refers to statements referring to defining good dementia care.
	Requirements of good dementia care	The code is used for statements referring to prerequisites for providing good dementia care. The code includes needs of/requirements for people with and without a migration background not only on an individual (person with dementia) or familial level, but also on a structural level.
	Role-Family	The code is used to extract statements about the role of family in providing good care for the person with dementia. The code includes statements regarding family responsibility and/or filial duty.
	Role_HC Personnel	The code stands for statements concerning the role of healthcare professionals in providing good care. The code includes statements regarding healthcare professionals’ responsibilities.
	Role-State	The code refers to statements concerning the role of state in providing good care. The code includes statements regarding state’s responsibilities.
Culture	Definition of culture	The code refers to statements where the participant describes what culture is or how the participant interprets/understands what culture is.
	Cultural attitudes	The code stands for statements about cultural attitudes, values or believes of people either with a Turkish or German origin. The code includes cultural attitudes of other nations, wherever possible.
	Cultural familiarity	The code refers to statements where the participant’s familiarity with a culture is mentioned/described. It includes positive or negative assessments and benefits of knowing/familiarity with a culture.
Challenges	Challenges for professionals	The code refers to statements about challenges that are faced by healthcare professionals and/or experts in the field.
	Challenges for families	The code refers to statements about challenges that are faced by families of people with dementia with or without a migration background. The code includes statements about psychological and/or physical burden, financial problems, problems in getting or finding support, etc.
	Challenges for TR people	The code refers to statements about challenges that are faced by people with migration background including specifically Turkish people living in Germany. This code includes challenges that might be linked to communication difficulties, such as language, barriers, such as education and income level, access problems, such as accessing and finding support groups and professionals (e.g., doctors, nurses, daily care), etc.
Integration	Integration	The code refers to statements about Turkish people’s integration to the German society. The code includes both negative and possible evaluations.
Culturally sensitive care	Culturally sensitive care	The code refers to statements about defining culturally sensitive dementia care and, where possible, its assessment in practice.
Individual centered care	Individual centered care	The code refers to statements about defining individual centered dementia care and, where possible, its assessment in practice.

## Results

The main findings of the study are presented below. For each theme, first the findings of the interviews with family caregivers with and without a migration background are presented, followed by the results of the interviews with the professionals. We present quotations for each theme to identify the similarities and differences among family caregivers with and without a migration background. Any differences and similarities among professionals’ perspectives with or without a migration background is also emphasized. The quotes are selected to illustrate the range of themes that emerged. We aim to highlight the ways in which the perspectives on and experiences with dementia care differ and overlap across different target groups by using such a comparative approach in presenting our findings.

### Perceptions of dementia influence access to information and support

A strikingly frequent point made by family caregivers with a Turkish migration background was the lack of knowledge about dementia and support services that are offered. If the interviewed caregiver does not have familiarity with dementia before, dementia and its symptoms were not recognized. As a result, symptoms often remain unrecognized for a long time. They often did not know where to receive important information.

I don’t know how much the nursing services take care of people with dementia. I don’t know exactly. I mean, if there were more information for us, for Turkish people, maybe that [would be good]. Otherwise, families are left on their own. […] If my parents were alone right now, I think they wouldn’t do anything. Nothing. Nothing at all. Turkish people don’t know [about the services] either. (Family caregiver, female, 2nd generation Turkish migrant)

In addition, dementia was described as being socially frowned and mixed up with mental insanity. Participants reported a fear of stigmatization within the Turkish community and therefore a desire to deal with the issue within the family only. The participants also reported social isolation, as the topic of dementia is generally hardly ever discussed.

My husband had so many mates and friends but they all left him. […] They labelled him crazy and abandoned him. When you are good you are very good, when you are bad no one knocks on your door. (Family caregiver, female, 2nd generation Turkish migrant)

This disease can completely upset a family. And [it is] culturally [relevant], for example, we [Turkish people] react against getting help from outside concerning care. (Family caregiver, female, 2nd generation Turkish migrant)

All family caregivers stated that physical, mental, legal and financial support is necessary when caring for a family member with dementia, as this is reported to be very challenging and burdensome. Some participants with a Turkish migration background cited a lack of cultural affinity and language barriers, as well as a sense of not being integrated into society, as reasons for their reluctance to ask for help and hesitation in seeking support.

I am especially glad that there are Turkish people. Because we, Turkish people, don’t really dare to go to the Germans and tell them or get help. It is easier for them [other members of the Turkish community with prior experience in dealing with legal and administrative matters] to understand us both linguistically and culturally. […] I mean, I feel that they would understand me more. (Family caregiver, female, 2nd generation Turkish migrant)

As a generational change is currently taking place and the person affected by dementia is the immigrant parent or grandparent, it was also reported that an increase in awareness is taking place. The family caregivers usually came to Germany as children or young people or were born in Germany. One participant reported the difficulty in making decisions about care, as he wanted to do the right thing in accordance to the cultural and traditional views of the older generation. The lack of knowledge and social incomprehension also leads to an overburdening of relatives with a Turkish migration background.

I have the feeling that people know far too little about dementia and how to deal with these people. My father doesn’t want people from the Turkish community to talk about mom that she is crazy and that’s why he doesn’t want any help with her care. I try to manage that, but I don’t have any time for myself anymore and that really drains me. […] Day care would help me a lot, but the family just doesn’t want that. (Family caregiver, female, 2nd generation Turkish migrant)

It was also reported that overburdening occurs when participants were assigned more tasks than they normally would, with the result of becoming unable to cope.

My husband spent three and a half years in a nursing home. But I went to the nursing home every day. […] I cooked him dinner, I washed him myself. […] I think I took very good care of him. They [the nursing home staff] were very pleased. Because I was doing their job. (Family caregiver, female, 2nd generation Turkish migrant)

In comparison, all participants without a migration background were quite aware of dementia and its associated symptoms. They described the progression of the stages quite neutrally and reported an immediate consultation with the family doctor after the onset of the first symptoms. Therefore, they could get an early diagnosis. The associated further measures accordingly started at a very early stage, such as applying for a level of care and the use of day care facilities.

When asking for access to information about dementia, self-help groups, information material on accommodation and help with health insurance applications, it became clear that participants without a migration background were much better informed. They usually obtained the information by asking their family doctor and carrying out additional searches by themselves. Nevertheless, the interviews revealed differences between participants from urban and rural areas. Those from rural areas reported experiencing social pressure from the village community and stigmatization associated with dementia. Even when participants were well informed, further action was often hindered by limited access to physicians and nearby support services. This issue was raised primarily by interviewees without a migration background; however, one participant with a Turkish migration background living in a rural area reported the same access barriers. This suggests that the same challenges can emerge in both rural and urban contexts, regardless of whether people have a migration background.

And I was also open about it [about my mother’s dementia and her need for support]here in the village. It wasn’t easy, they all looked at me. I said, ‘You all wear glasses [meaning that everyone relies on some form of support at times]^1^. You are just as much a form of support as this hospital stay was for my mother.’ And that helped. […] Here in the village, distances are long and when we’re not around, we have to rely on the local community. (Family caregiver, female, without migration background)

If you really have no one to help or drive you, and you want to place him [the person with dementia] in short-term care for a while, you have no option. […] Many children live far away, and by the time they get here, it’s also very exhausting if they have to keep coming all the time and I don’t drive anymore. (Family caregiver, female, without migration background)

The interviews with the professionals showed similar results. They stated that people with a migration background were very reluctant to ask questions about dementia, resulting in late diagnosis. This could also be due to language barriers, given that consultations usually took place with relatives and the medical history could only be obtained through translation. In addition, the lack of knowledge about support services among people with a Turkish migration background was linked by the professionals with a Turkish migration background to cultural attitude. They mentioned that Turkish people had a tendency to ignore dementia, preferred not to talk about the situation and took on tasks more than adequate, which also led people with a Turkish migration background to seek help at a very late stage. In comparison, people without migration background are mentioned to be more prudent, cautious and would like to take control at an early stage.

I think that Turkish families actually have a tendency to ignore dementia to some extent, to ignore the loss of cognitive abilities of the person with dementia to some extent, to try to compensate for the situation there, to do more work for them [the family members with dementia], but not to think too much that it might be dementia, [instead] attribute it to old age, and they can actually take on too many tasks. (Professional, male, 1st generation Turkish migrant)

Participating doctors and psychologists with a Turkish migration background reported a disproportionately high number of patients with a migration background. This is because people with a migration background either feel that they receive better medical care through communication in their mother tongue, or generally feel better understood in terms of cultural differences. However, this may also lead to mental and time-related overload for both families and professionals at the same time. When families with a migration background are in contact with professionals without a migration background, it is often necessary to clarify complex care situations and interdisciplinary issues, and to establish mutual understanding, which means consultations often take considerably longer. Furthermore, limited use of additional support services, such as mobile nursing services, may exacerbate the burden on existing care arrangements.

Especially among the first generation, dementia is also perceived differently in the Turkish language or culture. They don’t describe it as an illness but as mental madness. It is not so well regarded in society and therefore the disease is often concealed. Dealing with and accepting the illness is often difficult for relatives with a Turkish background, which is why I see different needs from the nursing and medical side. (Professional, male, 1st generation Turkish migrant)

I think many people [with migration a background] don’t even receive any help from third parties at home such as day care because they don’t even know that something like this exists or where to apply and receive information. And sometimes the understanding of the illness and symptoms is also very different. (Professional, female, without migration background)

### Managing care for people with dementia: the role of family and healthcare personnel

It turned out that cultural particularities affect the care choices of the family. With the exception of one participant^2^, all participants with a Turkish migration background took care of their relatives with dementia by themselves, mostly without the support of external care services. The desire and the duty to care for the family members with dementia was emphasized specifically by this target group. Many families said that they would prefer to care for their relatives at home. This was either because they did not have sufficient information about the support options available in nursing homes or because they did not think the services provided were good or adequate enough. The options of day care, financial support, support groups or short-term care were not widely used, partly due to unawareness and partly due to excessive bureaucracy and hurdles involved. In addition, some interviewees noted that even when they received financial assistance, it was still insufficient.

As long as he [WR5’s husband] stayed at home, they gave him, first of all, 235 Euros a month, because I looked after him. Not from the state, but from the healthcare insurance, I received care allowance for my husband. But there was nothing else. It was not enough. (Family caregiver, female, 2nd generation Turkish migrant)

Another point that was mentioned was that the options offered would not help because the person concerned would not be able to communicate with care staff. This could lead to unpleasant situations for the person concerned. The same explanation was given regarding placement in a nursing home. Most of this group of participants stated that in the nursing home, their relatives with dementia would be socially isolated, would not be able to sing along to German songs, would not know the games there and communication would be difficult. In addition, intimate care by third parties was also mentioned as a cultural and religious issue, which was perceived to be unacceptable to many people with a Turkish migration background.

I think that’s where two cultures collide. I’ve often heard from German friends here that, if it doesn’t work, mom and dad are sent to a nursing home, but not really with our people [people from Turkey]. Because as far as you can handle it, really do it. So, the parents stay at home and are cared for by the children. And I have to say, I - for me that wouldn’t be an option, so I would really care until the end. Well, maybe if they would specialize in dementia and have appropriate activities that suit also my parents with another cultural background, but we know nowadays in nursing homes they wouldn’t be in good hands. (Family caregiver, female, 2nd generation Turkish migrant)

I am the second generation here in Germany. And I’m the youngest in the family. I think I’m at a stage where you have to weigh things up a bit - the cultural aspects. Now they’re not yet in my position. And I did consider it [nursing home], but it was absurd, it’s your mom. How can you even think of considering something like that? (Family caregiver, male, 2nd generation Turkish migrant)

All family members without a migration background partly sought help from third parties. However, the living conditions and care situation varied greatly here compared to participants with a Turkish migration background. The participants without a migration background who cared for their relatives at home all made use of external help. Participants stated that receiving support from care services several times a week eased the burden of care and reduced the physical strain on relatives. They also applied for financial assistance in addition to payments related to the care degree, such as monthly sums for special equipment, chairs and similar. When asked where the knowledge about support services came from, the family doctor or community groups were usually mentioned. Nevertheless, the help offered was often described as inadequate and there was great dissatisfaction with the bureaucratic hurdles involved in offering help.

We have a nursing service involved. I just couldn’t do it on my own. I know a lot of money is needed, but […] we’re still fortunate enough to be able to pay for some things ourselves. But at some point, there’s a limit. (Family caregiver, male, without migration background)

Of course. Financial support. Well, what you get nowadays is ridiculous. […] You’re entitled to some euros; you’re supposed to find someone to help you. Whether that’s a qualified professional or not, it doesn’t matter to them. In that respect, you’re pretty much left on your own. (Family caregiver, female, without migration background)

All the other participants without a migration background had family members with dementia in nursing homes and were quick to decide against caring for themselves. Although there was some dissatisfaction with the nursing staff, there was a consensus that the situation had provided relief for the family and that they were generally satisfied.

You yourself are only able to cope to a limited extent and what use you have to the person being cared for if the person providing the care is no longer able to do so, if they themselves fall ill or if they no longer work. So, I think it’s really important to take the step [to place the family member with dementia in a nursing home]. (Family caregiver, female, without migration background)

All professionals with a Turkish migration background emphasized that within the Turkish community social pressure for taking care of the family member was so apparent. Usually taking care of the elder family members was seen as a filial duty. It could be the spouses or children, mostly it was the daughter or daughter-in-law, who could be exposed to social pressure. Whereas among people without a migration background, this was not the case. Quite the contrary, placing the family member with dementia in a nursing home was somehow expected.

When we talk about dementia, among Turkish families, the picture that usually emerges is that the family has a daughter, at least, and that daughter takes care of everything. […] It’s my mum or dad, it’s my filial duty, I have to do this. […] I think there is also some fear of social pressure here. You know the classic saying ‘what will people say?’, ‘oh look, he threw his mother there’. In order not to be exposed to such things, many people really feel such a social pressure [to take care of the family member]. (Professional, female, 2nd generation Turkish migrant)

The Germans have a very high respect for autonomy. They can say, ‘my father would not want it to be like this, for us to be exhausted for him. So let me put it in safer hands. Let me take him to a nursing home’, but it’s a bit different with us [people coming from Turkey], ‘what will people say?’ etc. (Professional, male, 1st generation Turkish migrant)

Participating doctors with a Turkish migration background reported to often take on the role of psychologists, especially for overburdened relatives who are unable to cope with full-time care.

Many come with memory problems of their relatives and then a conversation reveals severe depression. They are not treated psychiatrically or psychologically, they don’t have a therapist, they can’t find one. But these are things that they don’t want to discuss with family members or friends out of shame or whatever, and then the situation arises that they discuss it with me and sometimes for the first time in their lives. (Professional, male, 2nd generation Turkish migrant)

Professionals, specifically those who were nurses and social workers, also mentioned on the great difference in dealing with the care situations. They stated that this is mainly due to the fact that there are too few culturally sensitive care facilities available. Language barriers and cultural or religious differences were cited as the main barriers to access to support facilities. In addition, culturally sensitive facilities would mostly be used by people with a migration background, who are also better informed and have a higher level of education. However, some argued that it is not necessary to have a separate facility for this purpose, but rather to provide staff with training on the specific needs and expectations of people with a migration background.

The relatives of patients of Turkish origin are more likely to try to care for them at home. That’s their wish, that they look after them at home and care for them alone, but then they come to these difficulties afterwards, which they report, that a lot of things don’t work and they are overwhelmed, but out of respect for the family they still want to manage everything on their own. […] Unfortunately, they also often have little knowledge about support services or about dementia as a disease in itself. (Professional, male, 1st generation Turkish migrant)

### Good dementia care

The interviews showed interesting results regarding perceptions of good care. It became clear that this question is also culturally-influenced. While good dementia care was mostly addressed by professionals and the importance of nursing by the participants without a migration background, participants with a Turkish migration background talked more about family matters, maintaining and respecting the position of the affected person, respect for cultural circumstances and religious practices. The importance of cultural familiarity (e.g., language), financial support, understanding the person’s needs when receiving care was emphasized by all participants with a Turkish migration background. Even though all of them took care of the person with dementia at home, some mentioned institutionalization of the person with dementia would be good only if the care provided is culturally sensitive. Provision of good care was linked to trained, language-speaking nursing stuff.

We communicate in Turkish. For example, there is a German one [formal caregiver], she [the person with dementia] has a caregiver who comes from the health insurance. She [the formal caregiver] communicates with her in German, but she did not find her very warm or feel close to her. […] Language is a very big factor. She felt more comfortable with me. (Relative of a person with dementia, female, 1st generation Turkish migrant)

The desire to return to Turkey and receive care there was mentioned frequently, although legal hurdles were mentioned because care payments are no longer paid as soon the affected person is not in the Schengen area anymore. Four participants with a Turkish migration background mentioned that the question of returning to Turkey had already arisen and that care should maybe be sought there and would then be paid for by the family. One participant mentioned that the person affected had already lived in a nursing home in Turkey for 1,5 years, but due to the long distance, the affected person was back in Germany and was cared for alternately by family members.

Yes, there are differences. […] I think it’s very important to respond to the person’s habits and these habits differ, […] of course the language skills must be present on the therapeutic, nursing and medical side so that you can communicate accordingly. But there are more aspects [to good care] like knowing hygiene aspects, religious aspects, so that the patient feels comfortable. (Professional, male, 2nd generation Turkish migrant)

I would say it [culturally sensitive care] is about responding to the needs […] that you interact with the people, involve them as much as possible in communication, in social interaction, in their habits which then has the nice positive side effect that the people concerned are then simply calmer and yes, less irritable, run away less, become less aggressive, which at the end of the day, in my firm conviction, also leads to these people then also needing fewer neuroleptics, psychotropic drugs, other drugs to keep them calm. (Professional, male, 2nd generation Turkish migrant)

While participants with a Turkish migration background emphasized cultural familiarity, language, and religious considerations, some family caregivers without a migration background expressed shared concerns and priorities. For instance, a participant underlined the importance of care staff being sensitive and attentive to the needs of the person with dementia.

Some, like my mother, perhaps simply had fear of confronting the illness, but […] one or another conflict would not have had to arise if they had been better informed by good staff. So, I think education is very important. Education, training, and empathy of the staff. (Family caregiver, female, without migration background)

This view, expressed by a caregiver without a migration background, mirrors the emphasis on individualized, respectful care found in the accounts of caregivers with a migration background, even though cultural and linguistic aspects were less central in her narrative.

The interviewed professionals were in consensus that culturally sensitive care was an important factor in the integration of people with a migration background into care facilities as well as in their access to healthcare services in general. The aim is not to create separate facilities or access, but to encourage discussion about the needs and barriers faced by people with a migration background, and to break down the barriers by providing more information about support services. It was also stated that it is important to train nursing staff to be sensitive to cultural differences and to ensure that care facilities incorporate cultural needs.

When we talk about good dementia care, it’s about culturally sensitive care. Biographical background. In other words, a person’s family life, culture, education, religious traditions, customs and what they like in life. You need to know the biography, the life story of the person in order to provide quality service. […] So, when placing a person in a nursing home, you need to summarize their family life, where they were born, where they spent their life, where they grew old. And his values must be taken into account. (Professional, female, 1st generation Turkish migrant)

I know some Turkish care services in the districts where the proportion of immigrants is also higher. It works very well and is important for this generation, who barely understand German and are sometimes illiterate. On the other hand, unfortunately, I notice a resident of Turkish origin on my visits to the nursing home, who is visually striking, but is always alone. She looks very sad, always sits near the entrance and looks out. And I suspect that she has little personal contact with the other residents, perhaps due to the language barriers, and she may not be able to take part in the group activities that are offered in the nursing home. (Professional, female, without migration background)

It was emphasized that provision of good care is not limited to language, but very much related to cultural familiarity. It was stated that cultural familiarity helps to build trust between the professional caregiver and the person with dementia, resulting in better care and a greater sense of comfort. In dementia, reference to one’s culture was considered specifically important to provide good care. Culture understood as music, habits like body care and eating as well as religion. As with dementia care it gains much importance, because the person remembers only the things in the past.

Cultural means, that is, body care, clothing, worship, it covers all these things. These are important. Even the songs, music depends on the region they come from. That’s why we ask, where is this person from, where does he come from? We use music, music tones and sounds a lot. […] This is based on culture, for the comfort of that person. […] Maybe not in other diseases, but in dementia, since the disease is based on the person’s old life^3^, cultural situations are very important. (Professional, female, 2nd generation Turkish migrant)

By all professionals, the importance of support in the affected person’s mother tongue and stuff with cultural connotations were emphasized. Besides the emphasis on cultural background, person-centered care, that is care formed by person’s needs and with biographical background, became apparent.

Because people with dementia use their mother tongue. They do not use the language they learned later. Mother tongue, mother education, mother culture… They go back to the past. (Professional, female, 2nd generation Turkish migrant)

From the choice of meals to daily activities [cultural sensitivity is important in dementia care]. […] For example, in our dementia group, we sang beautiful Turkish art music songs. You know, it is necessary to do something like this. All these things relax the person with dementia, looking at old albums, old series’ music, old things, you know, songs from old Turkish films, etc. All these things can of course solve even behavioral problems. That’s why it is absolutely important. (Professional, male, 1st generation Turkish migrant)

### Diagnosing dementia in people with a migration background is a problem

The interviews revealed difficulties for people with a migration background to make a diagnosis using dementia tests at the general practitioner’s office, as dementia tests are in German and based on German familiar habits. Specifically participating doctors and psychologists stated that diagnostic tools for foreign-born people should be developed. The S-3 guidelines on dementia, for example, state that “the sole questioning of relatives about the function of the affected person has a similarly good diagnostic accuracy as a short cognitive test” [25]. However, the interviews revealed that cognitive short tests based on culturally specific (German) images and words cannot be used to diagnose people with a migration background, or can only be used incompletely. The validation of dementia tests cannot take place as it is related to language and education. This was mentioned as one of the reasons why foreign-born older people living in Germany are less likely to be diagnosed at an early stage, or more likely to be misdiagnosed, than the native population. Illiteracy in the older generation of migrants is also the reason why there is usually no diagnosis at all.

One important point is the diagnosis of dementia. Neuropsychological testing is validated for the German population. But now we have many illiterate people among the migrants, language barriers and other familiar everyday situations, so that most of the tests don’t work, even if you translate them. Maybe the patient can’t read, can’t do math, but doesn’t have dementia. Or he is severely demented and you are relying on the relatives’ story and making a misdiagnosis. (Professional, male, 2nd generation Turkish migrant)

Diagnostic tools are not very common. For foreigners, if I have to start with the diagnosis first, there is a problem in the validation of dementia tests for foreigners, because it is a linguistic thing and in order to test these cognitive functions, we need validation of instruments in a foreign language. […] Tools that diagnose language-independent dementia need to be further developed. […] It is also very education-dependent when you look at the validation of tests all over the world. Our elderly population [1st generation migrated from Turkey as guest workers] is unfortunately uneducated. I mean, you need at least eight years of education, for example, to validate a dementia test. (Professional, male, 1st generation Turkish migrant)

A German doctor cannot diagnose dementia very easily for a person who does not speak German. […] There is no option to measure dementia independent of language and culture. Therefore, those scales need to be developed first and then doctors need to be enlightened about this issue and they really need to undergo training in order to be able to use these scales easily. (Professional, female, 2nd generation Turkish migrant)

## Discussion

This study explored the perceptions of dementia and barriers to good dementia care that specifically affected people with a Turkish migration background in Germany. A key novel aspect of this study is its direct comparative design, which included family caregivers, as well as professionals, with and without a migration background. Participants without a migration background were deliberately included in order to distinguish migration-specific challenges in dementia care from more general issues, and to explore the cultural aspects of good, or effective, care for people with dementia. Considering these different groups enables a more nuanced interpretation of the findings and helps to avoid attributing general dementia care challenges solely to migration-related factors.

Our comparative analysis revealed how perceptions of dementia influence help-seeking behaviors for family caregivers with a Turkish migration background. For instance, the interviews with this group often revealed feelings of shame and stigmatization, as well as a lack of knowledge about dementia and how it progresses. These findings are consistent with those of other studies [3, 12]. A recent systematic review focusing on recruiting people with a Turkish migration background with dementia and their family caregivers as study participants also identified several issues, including cultural perceptions of dementia, a lack of knowledge about the disease and the relevant health services and system, and a lack of trust in doctors [26]. For caregiving relatives specifically, fear of stigmatization was also identified [26]. An earlier study observed the stigmatization and taboo surrounding the syndrome among people with a Turkish background, too [11]. These perceptions affect how people view health, illness and care. For instance, they may believe that dementia is simply a normal part of ageing, or that it is not an illness. Consequently, it may go unaddressed or be concealed [3, 4]. If the condition is not recognized as an illness, it is difficult to understand what is happening. Any action that could have been taken earlier, such as seeking help or treatment, is delayed, or the condition is ignored. The cultural stigma surrounding mental health issues can impact treatment-seeking behavior [27].

We found that care arrangements for family caregivers with a Turkish migration background were also culturally influenced. This brings about the role of family for taking care of their family member with dementia and making care decisions on their behalf. Cultural, religious and social norms significantly influence the preferred type of care, often resulting in people with a Turkish migration background choosing to care for their family member with dementia at home [3, 6, 11, 12, 22, 23, 26]. Similar to our findings, another study found that people with a Turkish migration background were less likely to use professional healthcare services [22]. The culture-value-laden conceptions of responsibility for elderly family members or filial duty reinforce the choice of family care over institutionalization of a family member with dementia[28]. This may also be related to how culturally shaped ideas about family responsibilities and the good life influence the care of people with dementia [4]. Thus, the effects of culturally shaped norms on care decisions can be demonstrated [4, 10].

We also identified structural gaps in the provision of good dementia care for people with a Turkish migration background. The requirements for improving care and quality of life for people with dementia and their families were similar for those with and without a migration background. Some difficulties, such as caregiver burden and bureaucratic barriers, were reported across groups in our study. Professionals described shared structural challenges in dementia care, such as limited resources and bureaucratic hurdles, but varied in their awareness of migration-related issues. However, the needs and expectations from healthcare personnel differed when caring for people with a Turkish migration background: while caregivers with a Turkish migration background emphasized cultural familiarity, language, and religious sensitivity, those without a migration background highlighted empathy, respectful interaction, and sufficient information. Nevertheless, they all agreed that good care should focus on each person’s individual needs and biographical background. All participating professionals emphasized these points, too.

The inadequacy of services is argued to be due to differences between dominant and non-dominant cultures, including linguistic, cultural, religious and ethnic differences [6, 29], but effectiveness is also dependent on professionals not being sensitive to cultural issues [13]. Considering dementia in particular, cultural sensitivity is becoming increasingly important in care [4]. Appropriate care for patients is best provided by healthcare personnel who take into consideration the patient’s cultural background and any culture-specific aspects. This means that healthcare services should be sensitive to different cultures [4]. Our findings highlight the importance of culturally sensitive care. Among all participating caregivers, only one person with a migration background was aware of an intercultural care facility, although it was not located in their own city. Nevertheless, there was considerable interest, and caregivers with a migration background emphasized that their concerns about institutionalized care would be alleviated if cultural aspects were taken into consideration. This finding is consistent with those of other studies (see [12]). Knowledge of and familiarity with the culture, customs, first language and biography of the person with dementia was identified by all participants as an important resource for providing good care to people with a migration background.

In addition to culturally sensitive care, a person-centered approach to dementia care is widely promoted in dementia care research. Inspired by Kitwood’s concept of care, which focuses on maintaining an individual’s sense of self (i.e., personhood) and well-being [30], this approach emphasizes the importance of considering an individual’s perspectives, personality, experiences, needs and their network of relationships when providing care [31, 32]. However, this approach would encourage a limited view of care. This is because belonging to a cultural group tends to become less significant when we consider that a person’s characteristics are shaped by their upbringing and living circumstances. To provide good care, it is crucial to identify the individual’s needs without ignoring the person’s cultural background^4^[33].

To further reflect on practical implications for aged care, hospital care and palliative care in Germany, we additionally sought the perspectives of two experts (not interview partners) with longstanding expertise in culturally sensitive healthcare, dementia care, and end-of-life ethics. Their reflections emphasized that culturally sensitive dementia care should remain strongly person-centered and avoid stereotypical assumptions about people with a migration background, as individuals differ considerably in their values, religious beliefs, preferences and expectations regarding care. In particular, individualized communication about care preferences, advance care planning, and end-of-life decision-making was highlighted as important in dementia care. Furthermore, the experts emphasized that sustainable implementation of culturally sensitive care requires structural and institutional approaches, including accessible information services, professional language mediation, culturally sensitive communication strategies, training opportunities for healthcare professionals and reflective supervision within healthcare institutions. Structural barriers may also influence the perception, accessibility and utilization of dementia-related healthcare services among people with a migration background. These considerations are in line with current discussions on culturally sensitive healthcare and communication in healthcare settings.

The major influence of language on access to dementia-related services, consultations with doctors and dealings and hesitation with care facilities is also reported from other studies. De Graaff et al. in this context describe language barriers and unfamiliarity with common care facilities in the Netherlands as the main reasons for a limited use of professional care services [34]. Our study also revealed that many participants with a Turkish migration background prefer to consult doctors who have a migration background by themselves. However, these doctors do not always share the same country of origin or language. This could be an indication that individuals with a migration background are concerned about being *othered* by doctors who do not have a migration background. When healthcare professionals and patients or their families have different cultural affinities, a process of othering may occur [27, 35]. If an action, behavior or request is deemed inappropriate or prejudiced by those in authority, such as healthcare professionals, it may be attributed to the service user’s country of origin or culture rather than being understood in the context of their individual circumstances [27]. This could result in a lack of trust in healthcare services, the perception of discriminatory behavior, and inadequate care for people with a migration background, which is unfair [4].

Communication problems pose a significant challenge for people with a migration background, with specific implications for the assessment of cognitive function and dementia diagnosis [36, 37] 5. The interviews with professionals in our study revealed communication and diagnosis barriers. Misdiagnosis or underdiagnosis can occur when testing and asking about German habits, language and writing. Conversely, people with a migration background may not be diagnosed due to shame, language barriers or misunderstandings. Studies indicate that the diagnosis rate for immigrant groups is lower than for the native population [39]. A variety of factors can influence behavior and the early diagnosis of dementia. These factors include a lack of trust in the healthcare system, fear of discrimination, low literacy and educational attainment levels, and the perception of dementia as a stigma rather than a medical condition. In addition to the language barriers and the accompanying feelings of shame experienced by the people with dementia and their relatives, potential medical complications, such as misunderstandings about diagnosis, medication, or treatment instructions, also emerge as a significant issue [37].

Language barriers may also partly be an issue of cultural differences leading to hesitation. In our study, family members with a Turkish migration background often felt misunderstood, believing that their cultural needs and wishes were not being addressed by nursing staff or doctors. According to the interviewed professionals, health and care must therefore be understood in a culturally diverse context to enable comprehensive support for people with dementia and their relatives. Şekercan et al. describe “cultural mismatches” in the context of issues encountered during consultations with healthcare personnel. They refer to cross-border healthcare — that is, seeking treatment or care in one’s country of origin, where language and cultural norms are more familiar — as a potential means of overcoming such barriers [40].

Various approaches can be taken to minimize access barriers to good care for people with a migration background. One approach is to make facilities more interculturally accessible. However, it must be considered whether these facilities also represent a form of segregation, potentially leading to a parallel healthcare system in the long term, rather than integrating the needs of minorities into the existing health care system.

Other approaches target at the widespread education of people with a migration background, the provision of information in socially disadvantaged areas with a high immigrant population, and the intercultural sensitization and training of healthcare staff. The practical guide issued by the nationwide working group on migration and public health describes this approach in detail [41]. Concept plans for the intercultural opening of healthcare institutions are presented, which include migration-specific aspects and thus aim to enable holistic care in the long term. Based on the findings of our study, such approaches are an important step towards reducing structural inequalities and ensuring equitable access to dementia-related healthcare services. Consistent and effective implementation of an intercultural approach to healthcare can significantly improve trust, communication and utilization among people with a migration background, promoting fairness and equal opportunities in care for all.

Based on our findings, several practical steps can improve culturally sensitive dementia care. First, communication should be adapted to the cultural and linguistic needs of patients and families. This includes using clear language, professional interpreters where necessary, and being sensitive to culturally shaped perceptions of dementia. Family members should be actively involved in care planning and decision-making, as familial responsibility plays a central role in many families with a Turkish migration background. Community-based and low-threshold services may help reduce barriers related to stigma, shame, and mistrust.

Our findings also highlight the need for structured training in intercultural competence for healthcare professionals in dementia care. Such training should focus on reflective skills, awareness of cultural differences, and strategies for building trust in care interactions. At the institutional level, policies that promote continuity of care, culturally sensitive information materials, and sufficient time for communication may help reduce feelings of shame and strengthen trust in healthcare services among families with a migration background.

## Limitations

There are some limitations to be considered for this qualitative interview study. First of all, the study participants do not fully represent the very heterogeneous group of people with Turkish migration backgrounds living in Germany. Nevertheless, care was taken to ensure that a broad spectrum was represented during the recruitment process.

During the recruitment process, we noticed that many people with migration backgrounds who were interested in the topic of the study nevertheless did not want to take part. The most common reasons given were fear of stigmatization, shame about disclosing their situation in dealing with a person with dementia, lack of time due to the very busy care situation, language barriers and fear of any possible negative consequences arising from participating in the study. These circumstances may have influenced the composition of our sample, as individuals who felt less affected by shame, stigmatization or fear of negative consequences were more willing to participate, potentially leading to an underrepresentation of more vulnerable or socially isolated perspectives within the study.

The professionals interviewed had specific experience of caring for and consulting with people with a migration background or dementia, which may have influenced their awareness of the challenges and potential solutions of intercultural dementia care. Nevertheless, this highlights the need for further sensitization and training of healthcare professionals, particularly those without prior intercultural experience, to better address migration-related and cultural barriers within the healthcare system.

We also noticed that participants with migration backgrounds who were recruited by ENY and did not have a good command of German wanted to conduct the interview in German although it was offered to conduct the interview in Turkish with ZAS. This highlights the importance of trust and personal connection in the recruitment process, as familiarity with the interviewer and informal, personal conversations significantly increased participants’ willingness to take part in the study and share their experiences more openly.

In general, the recruitment process proved to be very difficult and therefore tended to include people with migration backgrounds who were more open to the topic of dementia. Perspectives of people who are more hesitant to speak about the topic (be it on personal, cultural or other grounds) are therefore missing in the study.

## Conclusion

Equal access to health services should be provided to all. The strong discrepancy between the perception of dementia and lack of knowledge about healthcare services among people with a migration background, differences in the utilization of third-party help, as well as cultural and language barriers indicate a need for action. Culturally sensitive care plays an important role here, as well as the understanding of different needs and how to address them.

Our findings highlight ethical conflicts in the medical and nursing care of people with a migration background using the example of dementia care. As a consequence of our research findings, there are implications for a better integration of people with a migration background into the health system, which point to a necessary adaptation of the healthcare system as well as to further research on ethical conflicts and equality in medicine for all people living in Germany.

Culturally sensitive care does not mean stereotyping people with a Turkish migration background, but rather the integration of individual, cultural and religious needs in order to relieve the burden on relatives and care for people with dementia. The importance of culturally sensitive facilities and training for medical staff should be emphasized here. Especially with regard to the psychological and physical strain on relatives, it is important to have a holistic view of health and secondary diseases. Accordingly, culturally sensitive care should also be seen as a form of medical prevention and integration in order to enable holistic and long-term health promotion in society.

Such efforts in showing barriers for specific minority groups might help to raise awareness and contribute to improve access to healthcare services and good care for people with a migration background.

## Electronic Supplementary Material

Below is the link to the electronic supplementary material.

Supplementary Material 1

## Data Availability

Due to the sensitive nature of the research data, the data are not publicly available. Data may be available from the corresponding author upon reasonable request.

## Footnotes

1 The participant used a metaphor to normalize the need for support by referring to an everyday aid (wearing glasses), not to imply that older people are the same, but to frame receiving help as something ordinary.

2 This participant was no longer providing care herself. She stated that, at the request of her relatives, her family member with dementia had been placed in a care facility in Turkey.

3 The period until one is adolescent.

4 For a more detailed discussion on culturally sensitive care and person-centered care, please see [4].

5 For a detailed and comprehensive overview of the ethical challenges posed by the early diagnosis of dementia and predictive risk information, please see [38].
