# Supplementary material for: Expectations, realities and challenges of good dementia care for Turkish migrants in Germany: a qualitative interview study
Source: BMC Health Serv Res. 2026 May 15;26:710. doi: 10.1186/s12913-026-14728-3 (PMC13188498; doi:10.1186/s12913-026-14728-3)
Supplement: Supplementary file 1 — Supplementary Material 1 [file 12913_2026_14728_MOESM1_ESM.docx]

**Semi-Structured interview guidelines****^[[1]](#footnote-1)^**

**Stage 1: Semi-structured interviews with affected persons with and without migration background**

1. People who had experiences with taking care of family members with dementia and with migration background from Turkey
2. People who had experiences with taking care of family members with dementia and without migration background

**Biographical narrative stimulus**

- Please introduce yourself briefly and tell me why you wanted to participate in this study.
- I would like you to start by telling me about your life. When did you/your family come to Germany? Why?2
- Do you have a Turkish (people from Turkey) community here? How is your connection to them?2
- Do you have German friends, colleagues, acquaintances? How is your relation with them?^[[2]](#footnote-2)^
- How would you assess your integration with German society?2
- How do you assess your German language skills?2

**Perceptions about dementia and people with dementia**

- Have you ever cared for someone with dementia? If yes, could you please tell me about your experiences of taking care of your relative with dementia?

If not mentioned:

- - How caring for someone with dementia affected your private and social life?
- Please tell me about how it all began. How did you recognize that you/your relative have/has the signs of dementia? What was different or changed?
- What did you do when you recognized that your relative has the signs?
- What is your perception of the disease? How do you understand dementia? Has it changed following your personal experience?
- What do you think that other people think about people with dementia and their caregivers?

**Good dementia care**

- What do you understand by “good dementia care”? (What falls under?)
- What would contribute to the care and improve the quality of life of a person with dementia?

**The role of family for good dementia care**

- Please tell me a bit about your family (structure and relations).
- How is responsibility for caring for a family member with dementia shared in your family?
- Please consider the case that I am going to read to you now:

*Case Vignette (nursing home vs. taking care at home)*

*Mrs. A. migrated to Germany from Turkey. At the age of 65, Mrs. A. started to forget things and some daily tasks started to become difficult for her and as the time passed, she became more aggressive and her behaviors also changed. Her daughter took her to a memory clinic. The attending physician told the daughter that her mother has developed dementia. The doctor suggested to consider a nursing home as it would get more difficult. The daughter explained the doctor that he had a brother and that they could take care of their mother at home.*

- What would you do in this case? What do you think about it?
- Have you ever considered taking your family member with dementia to an institutional care facility (e.g., nursing home)? Why?
  - If not, why do you prefer to care for your family member with dementia at home?
- What challenges do you face in caring for a family member with dementia at home? Do you find it stressful? If so, in what way? What is stressful for you (if any) when caring for someone with dementia?
- What, if anything, does give you a sense of satisfaction when caring for your family member with dementia?

**The role of healthcare professionals in providing good dementia care**

- What are your expectations of healthcare professionals for the provision of good dementia care?
- What would you like to see changed or what would you wish to be avoided in order to receive proper care?
- Have you heard about advance care directives?

If not, explain:

- - In potential care options during the course of dementia, if the patient is unable to make a decision for himself/herself, his/her caring relatives or legally appointed proxies make the decision for the person. Advance directive would take effect at a time when the person is unable to express his or her own wishes.

If yes:

- - Who did provide this information to you and how?
- What do you think about advance care directives? Do you/your family member with dementia have one? Would you/your relative be willing to making one?

**The role of state in proving good care**

- Do you get any allowance from the state?
- What do you think should be done to improve the current situation?

**Experiences with and expectations from healthcare system**

- What has been your experience with the dementia-related healthcare services that your family member with dementia has received so far? Please describe any events and experiences that were important to you.
- Have you experienced any difficulties in clinical settings regarding the dementia-related healthcare services provided?
- In your opinion, what should be improved to utilize healthcare services for people with dementia?

**Are there any issues that have not been asked or talked about, but you would like to mention?**

**Semi-Structured interview guidelines**

**Stage 2: Semi-structured interviews with healthcare professionals with and without migration background**

**Biographical narrative stimulus**

- Please introduce yourself briefly and explain your professional background.
- Could you please tell me what your typical working day looks like? What makes a day good and bad for you at work?
- Do you have patients with migration background? Tell me about your experiences of providing care for people with migration background.
- Do you have any difficulties in interacting with people with migration background? If so, could you please describe?

**Perceptions of dementia**

- From your experience, do you think there is a difference between migrants from Turkey and Germans in terms of perceptions of the disease, attitudes towards the disease and the way people with dementia are treated?

**Good dementia care**

- What do you understand by “good dementia care”? (What falls under?)
- What do you think the requirements (prerequisites) are for good care for people with dementia?
- Do you think there are different requirements (prerequisites) to provide good care for older migrants from Turkey and support their families/caregivers?
- What do you think that needs to be improved in the healthcare system regarding the treatment and care of people with dementia with migration background?

**The role of family for good dementia care**

- Please consider the case that I am going to read to you now:

*Case Vignette (nursing home vs. taking care at home)*

*Mrs. A. migrated to Germany from Turkey in the late 1960s. At the age of 65, Mrs. A. started to forget things and some daily tasks started to become difficult for her and as the time passed, she became more aggressive and her behaviors also changed. Her daughter took her to a memory clinic. The attending physician told the daughter that her mother has developed dementia. The doctor suggested to consider a nursing home as it would get more difficult. The daughter explained the doctor that he had a brother and that they could take care of their mother at home.*

- What do you think about this case? In your opinion, is there a conflict here?

**The role of healthcare professionals in providing good dementia care**

- How is the setting when you disclose a diagnosis of early detection, MCI or dementia? Are the family members or other healthcare personnel involved in the setting?
- Could you explain the counseling process please? Do you offer a counseling session before and after disclosing a diagnosis or informing the person about her risk of developing the syndrome in the future? What does the consultation process include?
- During the counseling session, are the family members involved? How do you interact with them?
- At your institution, do you provide information on advance care directives?
- Do you guide the patients and their family members to prepare advance care directives? If so, how? If not, why?
- If the person has already developed dementia and does not have an advance care directive, how would you inform the family members concerning future treatment options and advance directives, for instance, in the sense of a proxy?

**The role of state in proving good care**

- What is the state’s responsibility (regulations, allowances and funding) in providing good care and support to the people with dementia and their families? What could/should be improved?

**Experiences of the treating physicians and attending healthcare personnel with people with and without migration background**

- Do you face any difficulties to provide care for people with dementia and their families? If so, could you please describe?
- Do you face any difficulties to provide care for people with dementia and with migration background? If so, could you please describe?
- Are there anything that you should pay attention to/ take into account when offering your services to people with migration background?
- If the person with dementia with migration background cannot (or would not prefer to) speak German anymore, how do you communicate? Do you have interpreters and mediators at your establishment? If not, do you approach to family members or do they approach to you? Do you see any issues here?
- What would help to improve the interaction with people with dementia with migration background and their families in clinical settings?

**Culturally sensitive care**

- What do you understand by culturally sensitive care/cultural competence in healthcare? How would you describe it?
- Do you see the importance of cultural competence/sensitivity in the care of person with dementia?
- Can you think of any specific challenges when providing care specifically for people with dementia with migration background?
- Did you have any training on cultural sensitivity or intercultural competency?
- What should be done to improve culturally sensitive care (i.e., care offered for people with dementia with migration background) in clinical settings on an organizational level?

**Are there any issues that have not been asked or talked about, but you would like to mention?**

1. We conducted the interviews in German and Turkish. For publication purposes, we translated the interview guidelines into English. [↑](#footnote-ref-1)
2. These questions are only asked to the family members or relatives of people with a Turkish migration background. [↑](#footnote-ref-2)
